# A novel step-by-step training program for transanal endoscopic surgery

**DOI:** 10.1186/s12909-023-04296-z

**Published:** 2023-05-11

**Authors:** Călin Popa, Diana Schlanger, Virgiliu Mihail Prunoiu, Ion Cosmin Puia, Florin Zaharie

**Affiliations:** 1 “Iuliu Haţieganu” University of Medicine and Pharmacy, Cluj-Napoca, Romania. Street Emil Isac no 13, Cluj-Napoca, 400023 Romania; 2 Surgery Department, Regional Institute of Gastroenterology and Hepatology “Prof. Dr. O. Fodor”, Street Croitorilor no 19-21, 400162 Cluj- Napoca, Cluj-Napoca, Romania; 3 “Carol Davila” University of Medicine and Pharmacy, Bucharest, Romania; 4 Clinic I General and Oncological Surgery “Prof. Dr. Alexandru Trestioreanu”, Bucharest, Romania

**Keywords:** Training program, Minimally invasive surgery, Transanal surgery, Surgical education

## Abstract

**Background:**

The objective of our study is to develop an effective training platform for transanal endoscopic surgery and to validate a step-by-step training program for learning the basic skills necessary for this approach.

**Methods:**

We have designed a two-part study: an experimental study (with the aim to design the training platform and the training exercises – on synthetic and biological material) and a prospective analytical study, in order to validate the training program by enrolling as participants general surgery residents and specialists, without previous experience in transanal endoscopic interventions. The performance of the participants was assessed based on the time of completion, as well as the quality of the execution.

**Results:**

We have developed three different diameter platforms (5 cm, 7.5 and 10 cm), that can be used with both the TEO and TAMIS platforms; specific exercises were developed to train different surgical skills like manipulation of tissue, cutting, dissection and suturing. Forty participants were enrolled for the validation of the proposed training program (12 young residents, 16 senior residents and 12 specialist surgeons). A statistically significant improvement of the performance time, from round to round, was observed for all participants in all exercises. The time of completion for the exercises, considering the correct technical execution, was the shortest for more experienced surgeons: specialist surgeons, followed by senior residents and young residents. The biological material exercises, that closely recreate intraoperative conditions and had more strict technical requirements, were difficult to be performed by young residents; better completion rates were seen in senior residents, while all the participants in the specialist surgeons group have completed these exercises.

**Conclusions:**

Our training program is an effective simulation based educational model for recreating intraoperative conditions particular to transanal endoscopic surgery. The proposed step-by-step training program has demonstrated to be useful in developing the important basic skills needed for transanal endoscopic surgery and assured the progress of all the participants, regardless of their surgical experience.

**Supplementary Information:**

The online version contains supplementary material available at 10.1186/s12909-023-04296-z.

## Introduction

The treatment of oncologic diseases, including rectal cancer, is continuously evolving, with a focus of not only curing the cancer, but also assuring a high quality of life. Transanal endoscopic microsurgery (TEM) was initially developed to assure improved surgical access to resect early-stage rectal cancer and benign rectal disease, with preservation of the morphological integrity and function of the organ [1–3].

Even though TEM registers important advantages, the downside of this technique is the steep learning curve. Several issues make TEM a challenging procedure: the restriction of movement on the horizontal axis, the reduced range of motion, the particularities of the surgical instruments and the difficulty of suturing the wall defect and manipulating the needle. Although none of these issues are definitive obstacles for TEM, it requires time to adapt to the new surgical conditions. Therefore, an intense educational program with hands-on training is necessary before applying this technique in clinical practice [4, 5].

Transanal endoscopic surgery has limited indications in well-selected patients, but its applications are continuously expanding, while its benefits are undeniable. From its early years, when it was used solely for the resection of small intraluminal masses, transanal surgery has now more diverse applications: from large local excisions, to major interventions like transanal total mesorectal excision (TaTME) [6, 7].

One of the barriers of the widespread introduction of TEM in surgical practice is the steep learning curve, translated through the fact that outcomes are highly influenced by the surgeon’s experience [8]; with increased experience, it was observed a reduction of the operating time, length of stay and complication rate [9, 10]. The need for dedicated training programs in order to improve the adoption and the outcomes of this technique has been stressed by several studies [5, 10, 11], and yet, even though several training methods have been cited, no structured step-by-step training program was reported [12]. While in some countries, the completion of formal TEM training is mandatory, many countries still do not have such requirements [5], partly due to the lack of availability of structured and reproductible training programs.

Our study intended to develop a training program that allows surgeons to start from basic exercises and to move on step by step to more complex techniques; subsequently, we have moved on to validate the respective training program by enrolling surgeons with different backgrounds in general and laparoscopic surgery and assessing their performance during the training period.

## Materials and methods

The present study consists of two parts. The first part is an experimental study, that aimed to develop a training system and different training exercises to aid the education of young surgeons in transanal endoscopic resections. The second part is a prospective analytical study that aims to validate the previously developed training program, by enrolling young surgeons and analyzing their progress.

### The development of the training systems and of the training program

#### The training systems

**Fig. 1:**
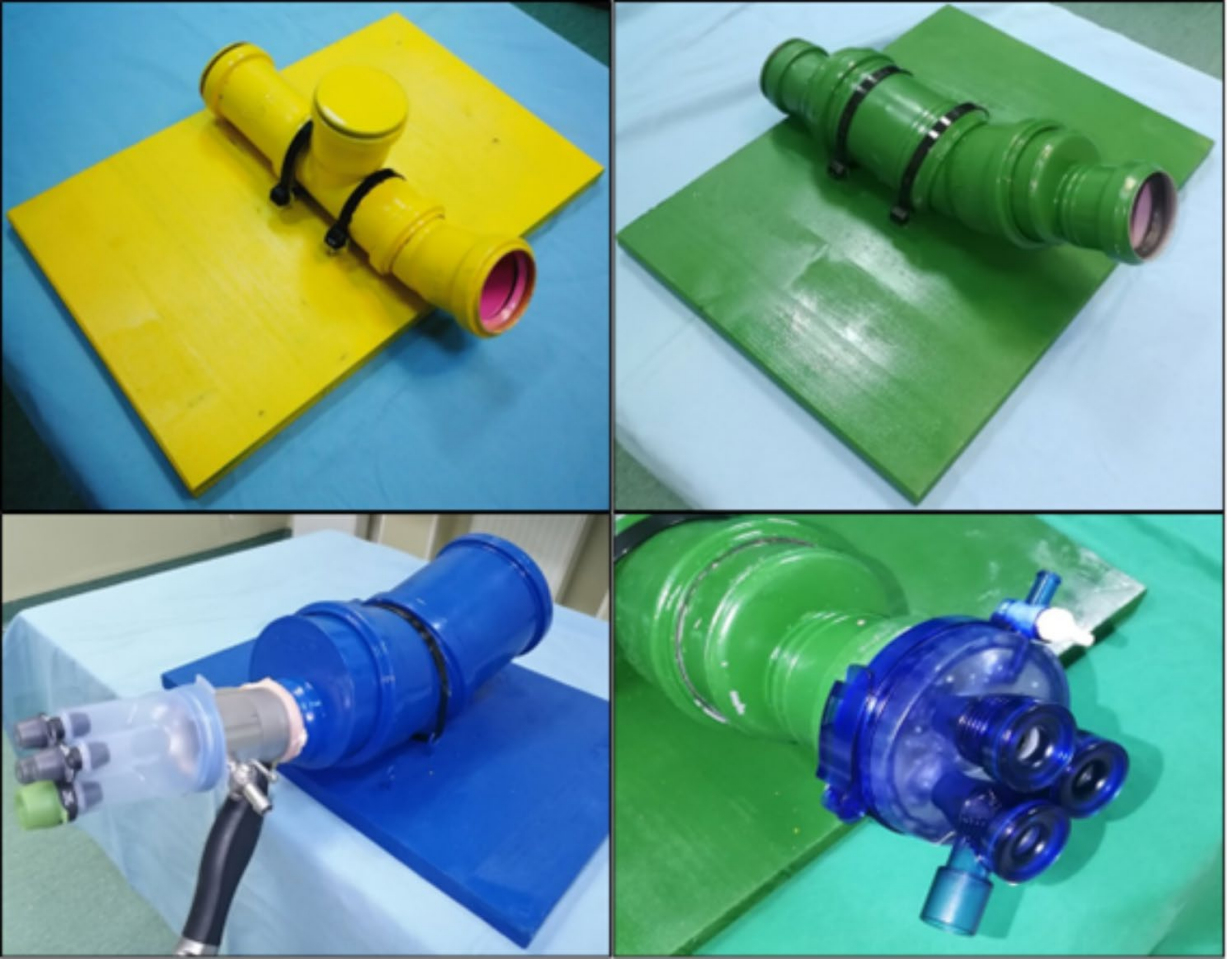
The designed tubular systems. 5 cm diameter (yellow), 7.5 cm diameter (green) and 10 cm diameter (blue)

A series of three tubular systems, with different diameters (5 cm, 7.5 and 10 cm respectively) have been designed in order to be compatible with either a transanal endoscopic operation platform (TEO) or a transanal minimally invasive surgery platform (TAMIS). The tubular systems are pictured in Fig. 1. The design intends to mimic the normal curvatures and diameters of a distended rectum. The tubular systems are detachable platforms fixed on a stable support, so they can easily allow adjustments inside the tubes, with an easy transition from one exercise to another. The 5 cm diameter tubular system is used only for synthetic material exercises, since it permits only a limited range of motion. The 7.5 cm diameter system is also used for synthetic material exercises, and in terms of range of motion, has more similarities to the human rectum. The 10 cm diameter system is used for exercises designed on biological materials. The swine stomach was used for this purpose, since it has several anatomical advantages, like a thick wall and clearly visible tissue layers so it can be used for submucosal dissection as well as for full-thickness resections. The stomach was prepared carefully with closure of the cardia in order to keep the insufflation of the organ, while the TEO or TAMIS platforms were fixed at the antro-piloric region (due to the anatomical characteristics that permit the use of the platform); this assembly should not pose problems and can easily be reproduced.

**Fig. 2:**
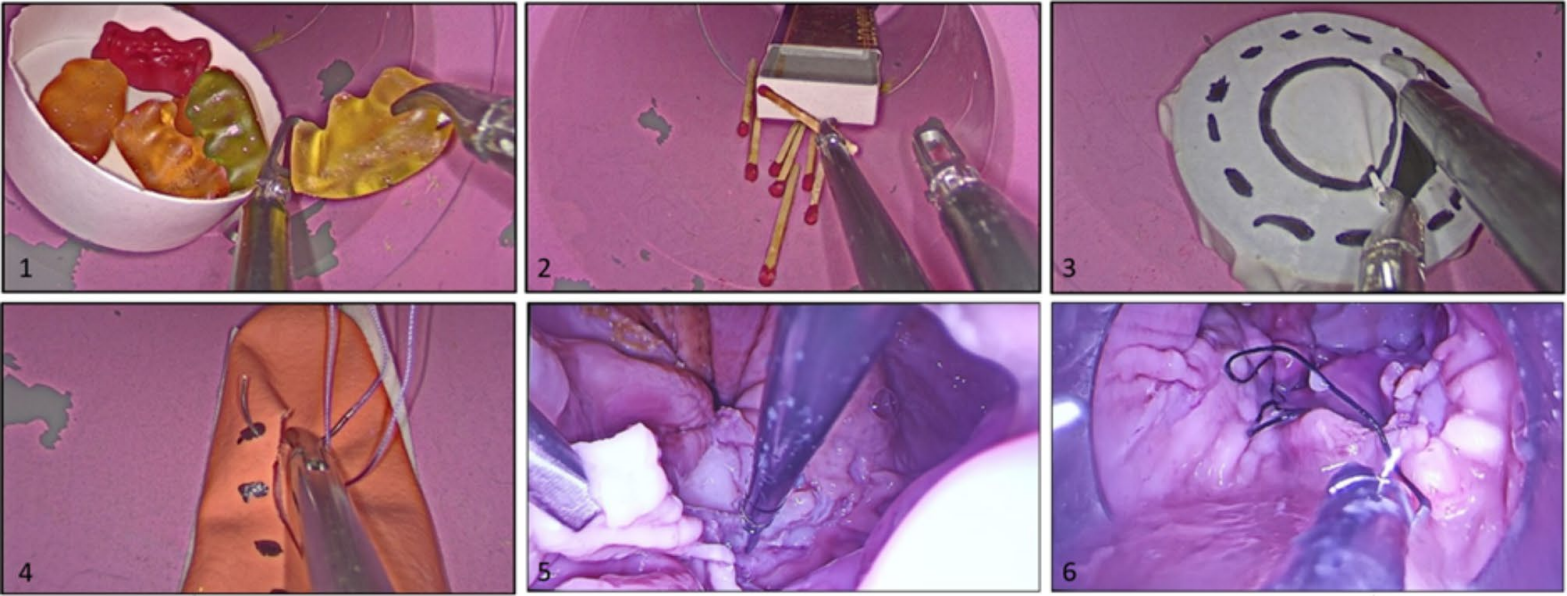
Training exercises

Two sets of exercises have been designed: one using synthetic materials and one using biologic material (swine stomach). The TEO platform (Karl Storz) or a gel-point TAMIS system (Applied Medical) were used, along with ordinary laparoscopic instruments (grasping and non-traumatic forceps, scissors, needle-holder). The tubular systems of 5 or 7.5 cm diameter were used for the synthetic material exercises, while the 10 cm diameter tubular system was used for the biological material exercises. Each exercise was timed: from the moment that the working instruments were inserted in the working ports, until the completion of the exercise and extraction of the instruments. The exercises are described in Table 1 and represented in Fig. 2

**Table 1:** The proposed exercises

Exercise name	Type of exercise	Materials needed	Description	Completion requirements	Tubular system (diameter)
Teddy bears	Synthetic	- Plastic recipient - 6 gummy bears - Two laparoscopic grasping forceps	Grasp one gummy bear at a time, transfer it from one forceps to the other and place it in the recipient.	Maximum 60 min/ 3 rounds	5 or 7.5 cm
Matches	Synthetic	- One empty match box, half opened. - 10 scattered matches - Two laparoscopic grasping forceps	The matches need to be introduced in the match box, assuring they have the same orientation (all matches with the tip in the same direction).	Maximum 60 min/ 3 rounds	7.5 cm
Beans	Synthetic	- A glove finger glued to a lateral wall of the tube. - 10 different size beans - Two laparoscopic grasping forceps	The beans need to be placed, one by one, in the glove finger.	Maximum 75 min/ 3 rounds	5 or 7.5 cm
Circles	Synthetic	- A latex sheet fixed in slight tension on a dedicated support with a drawing of two concentric circles with a few millimeters space between them. - A dissecting forceps and a scissor	The objective is to cut between these two circles, without touching the contour line.	Maximum 60 min/ 3 rounds	7.5 cm
Glove finger	Synthetic	- A rubbered glove finger, with a right cut on the superior surface, glued to the inferior wall of the tubular system. - A needle-holder, a dissecting forceps, and a scissor - A multifilament thread	The participant must introduce the suturing material in the tubular system, place a suture point and tie a triple knot, cut the thread, and extract the needle.	Maximum 60 min/ 2 rounds + an additional round if spare time	7.5 cm
Dissection and excision	Biological	- A non-traumatic forceps and scissor - Marked area on the swine stomach	A circumferential submucosal dissection and excision is undergone.	Two rounds – marked complete or not*	10 cm
Suturing	Biological	- A needle-holder, a dissecting forceps, and a scissor - Marked area on the swine stomach	The participant must introduce the suturing material in the tubular system, place a suture point and tie a triple knot, cut the thread, and extract the needle.	One round - – marked complete or not	10 cm

*In the first round the “lesion” was positioned on the inferior wall, while the second round increased the difficulty of the exercise by positioning the “lesion” in a more lateral position

1 – Teddy bears - The objective is to grasp one gummy bear at a time, transfer it from one forceps to the other and then, place it in the plastic recipient.

2 – Matches - Using two grasping forceps, the matches need to be introduced in the match box, assuring they have the same orientation.

3 – Circles – A dissecting forceps and a scissor are used. The objective of the exercise is to cut between these two circles, without touching the contour line.

4 – Finger glove – A suture point needs to be placed, tie a triple knot, cut the thread, and extract the needle.

5 – A circumferential submucosal dissection and excision is undergone.

6 –Biological material suturing– A suture point needs to be placed, tie a triple knot, cut the thread, and extract the needle.

### Part 2 – the validation of the training program

We conducted a prospective analytical study. The study population is represented by general surgeons and surgeons in training with a focus on colorectal surgery and a special interest in transanal surgery, but with no previous experience with this technique. Participants were enrolled through random sampling, between December 2020 and February 2021, by including them in three different hands-on workshops. The interested surgeons voluntarily registered for the events and afterwards underwent a selection process. General surgery residents and specialists were included. We excluded surgeons with experience in transanal endoscopic resections. Out of 63 registrations, 40 participants were selected based on the inclusion and exclusion criteria.

The training program consists in performing all the exercises described before, in a step-by-step manner. All exercises were timed, and the results were registered in a working sheet. During the hands-on workshops, the activity was supervised by 4 trainers. Before the start of the training program, each exercise was explained and demonstrated by a trainer. During the hands-on activity, the participants were supervised, cues and practical tips were given, and the participants had the opportunity to address questions; furthermore, the participants received feedback from the supervisors during and after the completion of each round. For an exercise to be considered complete, it had to respect the following conditions, hereby assuring the quality in the execution:

- Synthetic material exercises: an exercise was considered completed if the specific indications for the respective exercise were met, without causing any damage to the manipulated materials or instruments used.
- Biological material exercises: the biological material exercises had a greater emphasis on the quality of technique, the biological tissue allowing to evaluate and consider several additional points compared with the synthetic material exercises.
  - Dissection and excision – the participant needed to completely excise the marked area, while not excising unnecessary areas of “healthy tissue”, the dissection needed to be carried in the submucosal plane, without perforation and the surrounding margins of tissue should have minimal trauma and appear “viable”.
  - Suturing – the participant needed to place a suture point and tie a triple knot, while effectively adjusting the tissue margins and without harming the manipulated tissue.

After the completion of the workshop, the participants were contacted through a feedback form, that assessed the following points: the overall impression of the training program, their current practical activity regarding transanal surgery, the perceived advantages of undergoing a structured training program. The feedback form was anonymous.

The study has been approved by the Ethics Committee of Iuliu Hatieganu University of Medicine and Pharmacy Cluj-Napoca (Number 310/2021). All methods were carried out in accordance with relevant guidelines and regulations.

Data analysis was performed using R 3.5.1. Normality of the distribution was assessed using Shapiro-Wilk’s test and histogram visualization. In this direction, we also considered the small number of individuals included and the number of individuals per group and decided to use a non-parametric approach. Categorical variables were represented using absolute value (percent). Contingency tables were assessed using Fisher’s exact test. Continuous data was represented as median (quartile 1, quartile 3). Differences between two paired non-normally distributed variables were assessed using Mann-Whitney-Wilcoxon signed rank test. Differences between two non-paired non-normally distributed variables were assessed using Mann-Whitney-Wilcoxon rank sum test. For comparisons where summing of the three rounds had to be performed, the participants who did not finish the test were given a score of 75 (the maximum -number of minutes considering all tests). Given the rank-based algorithm of non-parametric tests, this approach was used to estimate the ranks of the participants. A p-value under 0.05 was considered statistically significant.

## Results

### Part 1 – the development of the training systems and of the training program

The first part of the study consisted in developing the three tubular systems and the 7 exercises previously described in the Methods section. The platforms were built using usual household materials, in order to be compatible with either a TEO or a TAMIS platform. The approximate cost of building a platform was 60 euros. We aimed to develop exercises that helped practice different skill sets:

- Tissue manipulation: ‘Teddy Bears’, ‘Matches’, ‘Beans’.
- Cutting and dissection: ‘Circles’, ‘Biological material dissection and excision’.
- Suturing: ‘Glove finger’, ‘Biological material suturing’.

### Part 2 – the validation of the training program.

For the second part of the study, we enrolled 40 participants, that were divided in three groups based on their surgical experience:

- Group A: young residents (years 1 to 3 of training): 12 participants.
- Group B: older residents (years 4 to 6 of training): 16 participants.
- Group C: senior surgeons: 12 participants.

The times of completion of each participant for each round, for the synthetic material exercises, can be seen in a table in the Supplementary Material; another table attached as well in the Supplementary Material, details the exercises done on biological materials, noting “Yes” or “No” if the participant has completed or not the respective round. In Table 2, we have synthetized the average time and the completion rates of each round, on the overall cohort of participants as well as separately, on the three subgroups.

**Table 2:** Average time and completion rates of the proposed exercises

		TEDDY BEARS 5CM			TEDDY BEARS 7.5CM			MATCHES 7.5CM			BEANS 5CM			BEANS 7.5CM			CIRCLES 7.5CM			GLOVE FINGER 7.5CM		
		1	2	3	1	2	3	1	2	3	1	2	3	1	2	3	1	2	3	1	2	3
A	Average time (min)	21.3	15.7	8.0	17.3	14.3	6.3	27.0	19.7	14.5	36.3	26.9	17.7	34.0	25.6	19.0	20.3	15.8	7.5	27.8	20.4	8.0
	Completion rate (%)	100	100	33.3	100	100	58.3	100	100	16.7	100	91.7	25.0	100	91.7	25.0	100	100	50.0	100	83.3	8.3
B	Average time (min)	14.0	10.1	7.4	12.4	8.7	6.9	21.9	16.3	11.4	28.2	21.9	16.2	28.0	21.3	16.0	15.4	11.5	6.3	18.3	14.3	7.5
	Completion rate (%)	100	100	100	100	100	100	100	100	87.5	100	100	68.8	100	100	81.3	100	100	93.8	100	100	62.5
C	Average time (min)	10.2	6.3	3.6	8.3	5.1	2.8	13.7	8.6	6.4	22.6	12.7	10.0	20.6	11.4	8.4	9.9	5.8	3.3	11.5	6.9	3.8
	Completion rate (%)	100	100	100	100	100	100	100	100	100	100	100	100	100	100	100	100	100	100	100	100	100
Overall	Average time (min)	15.0	10.6	6.1	12.6	9.3	5.4	21.0	15.0	9.5	29.0	20.5	13.5	27.6	19.4	13.1	15.2	11.1	5.4	19.1	13.6	5.6
	Completion rate (%)	100	100	80.0	100	100	87.5	100	100	70.0	100	97.5	65.0	100	97.5	70.0	100	100	82.5	100	95.0	57.5

### Synthetic material exercises

The progress from one round to another was shown through a statistically significant improvement of the performance time for all exercises (p < 0.01). The time improvement was also statistically significant when analyzing it on the three different subgroups (p < 0.01). For Group A, due to the small completion rates of round 3, analyzing the progress from round 2 to round 3 was not relevant.

**Fig. 3:**
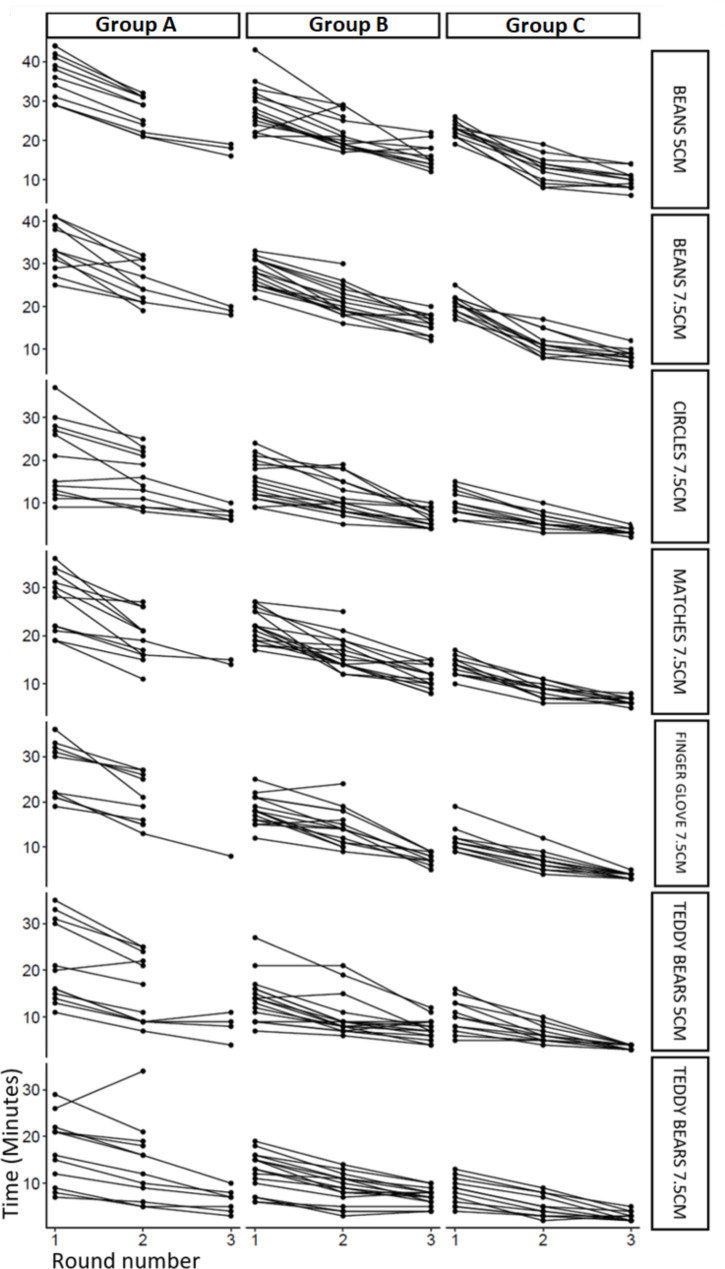
The evolution of the study participants

Group C has registered shorter times (a faster execution of the exercise) than group B and A, while group B registered shorter times than group A (p < 0.01). Figure 3 shows the evolution of the participants through the proposed exercises.

Based on the time of completion for each exercise, it was observed that the ‘Teddy bears’ and the ‘Circles’ exercises are of similar difficulty (p = 0.17), while the ‘Matches’ and the ‘Glove finger’ exercises are as well of similar difficulty (p = 0.58). The ‘Teddy bears’ and the ‘Circles’ were the least difficult to perform, followed by the ‘Matches’ and ‘Glove finger’; the ‘Beans’ were the most difficult exercise to perform, with the longest completion times (p < 0.01). The same exercise performed on different diameter tubular systems (‘Teddy bears’ and ‘Beans’) did not have a statistically significant difference between them (p = 0.12 and p = 0.58), therefore they can be considered of similar difficulty.

The figure illustrates the performance time of each participant, stratified by Group and Exercise. Improvement in the times of completion can be observed for most of the participants, as the rounds advance.

### Biological material exercises

Round 1 of the dissection exercise was 100% completed. Taking into consideration the quality criteria described in the Methods section, the completion rate of round 2 of the dissection exercise and the suturing exercise was low for group A (16.7% and 0%), higher for group B (75% and 56.3%), while for group C, all participants have completed the exercises (100% and 100%).

### Feedback from the participants

Thirty-one responses were received (77.5%). Regarding the overall impression of the training program, the responses were either ‘Good’ (25.8%) or ‘Very good’ (74.2%). Regarding their current practical activity in transanal surgery, 93.5% reported a higher involvement in cases compared to before undergoing the training program. The most indicated perceived advantages of undergoing a structured training program were a better understanding of the working space, better camera navigation skills and familiarization with the types of movements specific to the technique.

## Discussion

Transanal minimal invasive surgery is an innovative approach for the treatment of several rectal diseases,, with indications that are expanding continuously, making it an integrative part of surgical practice [13–15]. However, this technique brings important technical limitations: a challenging technique and a steep learning curve [16, 17].

Conversion rate, procedure time, as well as postoperative complications are correlated with the learning curve of this technique [10]; improved outcomes are observed after around 20 cases [18, 19]. Therefore, the clinical need of a structured and reproductible training program is clearly outlined and the implementation of such a program might improve learning curves among surgeons.

The rapid advancement of surgical technologies has mandated changes in the traditional apprenticeship model of training with simulation-based educational methods being more and more adopted in diverse fields. Simulation has the great advantage of allowing the trainees to practice and rehearse in a controlled environment, with special focus on their needs [20, 21] and has been proven to have great benefits in laparoscopy training [22–24]. While there is a high variety of simulation methods, the high costs are still a major impediment [20, 21]. Our proposed training model follows the principles of simulation education, while assuring a low cost of implementation. Considering the features of a high-fidelity simulator [25], our model assures that learners can engage in a repetitive practice, perform exercises with different levels of difficulty, with a clear outline of the expected outcomes under supervision with feedback from trainers; furthermore, our model encourages individualized training, in a controlled environment that can capture different variations in scenarios and the model can be easily adapted to multiple learning strategies. Since our proposed model manages to recreate intraoperative conditions with fidelity in terms of visuospatial perceptions, types of movements and tasks, the model offers a good simulator validity. However, curriculum implementation remains a downside, but we believe that the reproducibility of our training program creates favorable conditions for including this model in the educational curriculum of surgical residents and colorectal specialist surgeons.

Information regarding training programs for minimally invasive transanal surgery is scarce in the medical literature. However, several recommendations dictate that it is advisable to undertake training before safely implementing this procedure in clinical practice [12]. Laparoscopic surgery training programs have been proven to be useful and highly needed to assure a proper education for young surgeons [26–28], which also highlights the need and utility of such training programs specially designed for transanal minimal invasive surgery. We have performed a literature search that did not identify any other similar training programs to ours. Therefore, we believe that our study solves an important gap in the training of colorectal surgeons. Although an initial training program was proposed with the release of the first TES platforms, it offered insufficient details and did not meet the needs of training the currently used techniques in transanal surgery [29]; some other studies aimed to develop training simulators for teaching TaTME [30–32]. Even though our proposed program does not go as far as teaching TaTME, we believe that a proper training in the first steps of transanal endoscopic surgery are essential for establishing a good base before learning more advanced techniques.

Our proposed training program provides a systematic approach to learning the necessary skills for transanal endoscopic surgery. We have developed several exercises on synthetic materials, that allowed the participants to develop tissue manipulation skills (grasping different materials, with different textures with the forceps and performing different basic tasks: ’Teddy bears’, ‘Matches’ and ‘Beans’ exercises), dissection skills (cutting a certain material based on a previously drawn sketch, by using a forceps and a dissecting scissor: ‘Circles’ exercise) and suturing skills (using the needle-holder, manipulating and arranging the needle and placing the suture point and tying the knot: ‘Glove finger’ exercise). Some exercises (’Teddy bears’ and ‘Beans’) were performed on 2 different diameter tubular systems – since there has been no significant difference in performance of the same exercise on different platforms, a refined training program could only include the practice of all synthetic exercises in the 7.5 cm diameter system, eliminating the need of an additional platform. The training program goes one step further, by more accurately replicating the intraoperative conditions, through specific exercises on biological material, that simulates the dissection with excision of a certain lesion and the suturing of the rectal walls. Even though the focus of our analysis was on the performance time, the completion of the exercise was conditioned by respecting some quality criteria; therefore, the completion of an exercise implied a correct execution of the targeted skill. Especially in the case of the exercises on biological material, the focus was shifted from the time of completion to the correct execution of the task; the exercises being only noted as completed or not, considering the quality of the execution.

As we can see from the presented results, the progress of all participants was evident from one round to another: this shows that practice improves orientation in the narrow workspace particular to transanal endoscopic surgery, familiarizes the participant with the used instruments and teaches different skills necessary to perform a safe and efficient intervention. The progress has been demonstrated for all included participants, therefore proving the effectiveness of the training program.

The more experienced surgeons, as expected, performed the proposed exercises better; this underlines the fact that transanal minimally invasive surgery, although it has certain differences with laparoscopic surgery, still uses the same principles and being proficient in laparoscopy helps in the training process. However, the training program is beneficial even for senior surgeons, as seen from the presented results.

The exercises performed on biological material, that replicate most accurately the intraoperative conditions, seem to be too difficult for young surgeons (group A). Therefore, we believe that in the future, the training program should be defalcated for separate categories: for category A, we recommend synthetic material exercises, for category B we believe that introduction of the biological material exercises is useful, while for category C the focus should be set on the biological material exercises.

The novelty of our study consists in reporting the only systematic step-by-step training model for transanal endoscopic surgery in the medical literature, that has a high reproducibility, and consists of standardized exercises with targeted training of essential skills. One of the biggest advantages of the program that we proposed is its feasibility, due to the low costs of building the training platforms and the fact that they are made of largely available household materials. Therefore, these platforms can be widely used even in low-income countries, in centers that may not afford to send surgeons to expensive training courses. On the other hand, the presented exercises are easy to reproduce; they target all the important skills for performing an effective minimally invasive transanal procedure and they have clear and easy-to-follow instructions. Nonetheless, the present study proves that this basic training program is effective for different categories of surgeons, from residents to senior surgeons, and we have demonstrated the improvement of the targeted skills.

Even though our program was shown to be effective, it is unclear how long the trained skills will be maintained. Rehearsal is essential, so the implementation of such a program in the training curriculum of young surgeons could be a way to assure continuous medical education and maintenance of skill level. Regarding the translation of the trained skills in clinical practice, the participants’ feedback showed positive results, which should encourage the continuance of the program.

Our program focused on training the basic skill level for performing safe and effective transanal minimally invasive interventions. Ideally, our proposed program should be included in a more complex training program, with a focus on continuous medical education and should serve as a steppingstone before starting these types of interventions in clinical practice and before pursuing more advanced techniques, including TaTME or the treatment of perirectal neoplasms [33, 34].

Our study has of course its limitations: it is a pilot study with a relatively small number of participants, without randomization of the participants, that presents and discusses only our proposed training program, since no similar training programs were reported in the medical literature. It is worth noting as well that initiating the program during the pandemic has made the enrollment of participants more difficult; however, we plan a continuity for the program to prove its validity on a larger sample. Another issue that needs to be addressed by further studies should be the maintenance of the acquired skill level over time, by following the participants’ clinical activity and reassess their skills through another simulation-based program.

## Conclusion

A well-structured training program is useful and efficient for surgeons that intend to start performing minimally invasive transanal surgeries. We have designed a training program for basic skills in transanal endoscopic surgery, that has been validated in both surgical residents and senior surgeons. Our proposed step-by-step program targets all the important skills needed in such interventions and it has been demonstrated that assures the progress of the surgical skills of the participants.

## Electronic supplementary material

Below is the link to the electronic supplementary material.

Supplementary Material 1

Supplementary Material 2

## Data Availability

The datasets are available as Supplementary Material.
