# Supplementary material for: A novel step-by-step training program for transanal endoscopic surgery
Source: BMC Med Educ. 2023 May 11;23:327. doi: 10.1186/s12909-023-04296-z (PMC10176938; doi:10.1186/s12909-023-04296-z)
Supplement: Supplementary file 1 — Supplementary Material 1 [file 12909_2023_4296_MOESM1_ESM.docx]

Table 2. Biological material exercises

|  | DISSECTION 12CM | | SUTURE 12CM |
| --- | --- | --- | --- |
|  | Round 1 | Round 2 |  |
| A1 | Yes | No | No |
| A2 | Yes | No | No |
| A3 | Yes | No | No |
| A4 | Yes | No | No |
| A5 | Yes | Yes | No |
| A6 | Yes | No | No |
| A7 | Yes | No | No |
| A8 | Yes | No | No |
| A9 | Yes | No | No |
| A10 | Yes | No | No |
| A11 | Yes | Yes | No |
| A12 | Yes | No | No |
| B1 | Yes | Yes | No |
| B2 | Yes | No | Yes |
| B3 | Yes | Yes | Yes |
| B4 | Yes | No | Yes |
| B5 | Yes | Yes | No |
| B6 | Yes | Yes | Yes |
| B7 | Yes | Yes | Yes |
| B8 | Yes | No | No |
| B9 | Yes | Yes | Yes |
| B10 | Yes | Yes | Yes |
| B11 | Yes | Yes | No |
| B12 | Yes | Yes | Yes |
| B13 | Yes | No | No |
| B14 | Yes | Yes | No |
| B15 | Yes | Yes | No |
| B16 | Yes | Yes | Yes |
| C1 | Yes | Yes | Yes |
| C2 | Yes | Yes | Yes |
| C3 | Yes | Yes | Yes |
| C4 | Yes | Yes | Yes |
| C5 | Yes | Yes | Yes |
| C6 | Yes | Yes | Yes |
| C7 | Yes | Yes | Yes |
| C8 | Yes | Yes | Yes |
| C9 | Yes | Yes | Yes |
| C10 | Yes | Yes | Yes |
| C11 | Yes | Yes | Yes |
| C12 | Yes | Yes | Yes |

* The unit of time for all the results presented in this table is ‘minutes’
