# Supplementary material for: A novel step-by-step training program for transanal endoscopic surgery
Source: BMC Med Educ. 2023 May 11;23:327. doi: 10.1186/s12909-023-04296-z (PMC10176938; doi:10.1186/s12909-023-04296-z)
Supplement: Supplementary file 2 — Supplementary Material 2 [file 12909_2023_4296_MOESM2_ESM.docx]

Table 1. Synthetic materials exercises

|  | TEDDY BEARS 5CM | | | TEDDY BEARS 7.5CM | | | MATCHES 7.5CM | | | BEANS 5CM | | | BEANS 7.5CM | | | CIRCLES 7.5CM | | | GLOVE FINGER 7.5CM | | |
| --- | --- | --- | --- | --- | --- | --- | --- | --- | --- | --- | --- | --- | --- | --- | --- | --- | --- | --- | --- | --- | --- |
| Round | 1 | 2 | 3 | 1 | 2 | 3 | 1 | 2 | 3 | 1 | 2 | 3 | 1 | 2 | 3 | 1 | 2 | 3 | 1 | 2 | 3 |
| A1 | 14 | 9 |  | 8 | 5 | 5 | 19 | 15 |  | 34 | 25 |  | 29 | 31 |  | 11 | 11 | 6 | 21 | 15 |  |
| A2 | 15 | 11 |  | 9 | 5 | 3 | 22 | 17 |  | 36 | 29 |  | 31 | 22 |  | 9 | 9 | 7 | 22 | 13 | 8 |
| A3 | 11 | 7 | 4 | 7 | 6 | 4 | 19 | 11 |  | 29 | 21 | 16 | 25 | 21 |  | 13 | 8 | 6 | 22 | 19 |  |
| A4 | 13 | 9 | 8 | 12 | 9 | 7 | 21 | 19 | 14 | 29 | 21 | 18 | 27 | 21 | 18 | 12 | 9 | 8 | 19 | 16 |  |
| A5 | 16 | 9 | 11 | 15 | 10 | 8 | 22 | 16 |  | 31 | 24 |  | 33 | 24 | 19 | 14 | 13 | 8 | 21 | 15 |  |
| A6 | 21 | 17 |  | 16 | 12 | 7 | 30 | 21 |  | 41 | 31 |  | 32 | 19 |  | 15 | 16 | 10 | 31 |  |  |
| A7 | 30 | 21 |  | 21 | 19 |  | 34 | 26 |  | 44 |  |  | 39 |  |  | 21 | 19 |  | 36 |  |  |
| A8 | 35 | 25 |  | 22 | 16 |  | 31 | 26 |  | 44 | 31 |  | 39 | 24 |  | 26 | 14 |  | 30 | 27 |  |
| A9 | 20 | 22 |  | 21 | 16 | 10 | 28 | 27 |  | 39 | 31 |  | 41 | 29 |  | 27 | 21 |  | 32 | 25 |  |
| A10 | 16 | 9 | 9 | 26 | 34 |  | 33 | 21 |  | 29 | 22 | 19 | 33 | 27 | 20 | 28 | 22 |  | 36 | 21 |  |
| A11 | 33 | 24 |  | 29 | 21 |  | 36 | 21 |  | 38 | 29 |  | 41 | 32 |  | 30 | 25 |  | 33 | 27 |  |
| A12 | 31 | 25 |  | 21 | 18 |  | 29 | 16 | 15 | 42 | 32 |  | 38 | 31 |  | 37 | 23 |  | 31 | 26 |  |
| B1 | 9 | 9 | 6 | 7 | 4 | 4 | 17 | 14 | 10 | 32 | 22 |  | 25 | 19 |  | 9 | 10 | 5 | 18 | 10 |  |
| B2 | 9 | 7 | 5 | 6 | 5 | 5 | 18 | 16 |  | 28 | 19 | 14 | 24 | 19 | 13 | 12 | 8 | 5 | 17 | 10 |  |
| B3 | 11 | 8 | 4 | 7 | 3 | 4 | 21 | 14 | 9 | 30 | 21 |  | 25 | 21 |  | 9 | 5 | 4 | 15 | 16 |  |
| B4 | 7 | 6 | 4 | 6 | 4 | 4 | 20 | 14 | 8 | 27 | 18 | 14 | 31 | 19 | 15 | 11 | 7 | 4 | 18 | 14 | 8 |
| B5 | 9 | 7 | 6 | 11 | 9 | 6 | 19 | 18 | 10 | 21 | 21 | 15 | 25 | 20 | 15 | 11 | 8 | 6 | 12 | 9 | 7 |
| B6 | 12 | 8 | 7 | 12 | 11 | 6 | 18 | 17 | 12 | 22 | 29 | 15 | 28 | 19 | 16 | 12 | 10 | 9 | 16 | 14 |  |
| B7 | 13 | 7 | 9 | 10 | 7 | 8 | 22 | 15 | 14 | 26 | 19 | 13 | 27 | 18 | 18 | 16 | 11 | 9 | 21 | 18 |  |
| B8 | 15 | 8 | 7 | 13 | 9 | 7 | 25 | 12 | 11 | 33 | 29 |  | 29 | 21 | 17 | 13 | 8 | 4 | 18 | 11 | 7 |
| B9 | 14 | 9 | 9 | 15 | 8 | 8 | 22 | 19 | 14 | 24 | 20 | 18 | 26 | 18 | 12 | 14 | 9 | 4 | 15 | 14 | 5 |
| B10 | 16 | 9 | 8 | 13 | 8 | 7 | 19 | 14 | 8 | 22 | 17 | 18 | 22 | 16 | 13 | 15 | 10 | 5 | 16 | 12 | 6 |
| B11 | 21 | 21 | 11 | 16 | 11 | 8 | 25 | 21 | 15 | 43 | 28 |  | 31 | 25 | 16 | 22 | 13 | 10 | 18 | 11 | 9 |
| B12 | 14 | 15 | 7 | 19 | 14 | 10 | 22 | 14 | 15 | 26 | 19 | 21 | 28 | 22 | 18 | 18 | 19 |  | 21 | 14 | 8 |
| B13 | 14 | 8 | 9 | 16 | 13 | 10 | 27 | 25 |  | 31 | 25 | 22 | 31 | 23 | 18 | 19 | 18 | 8 | 25 | 19 | 9 |
| B14 | 27 | 19 | 12 | 15 | 11 | 9 | 26 | 19 | 12 | 35 | 26 |  | 33 | 30 |  | 24 | 15 | 8 | 22 | 24 |  |
| B15 | 17 | 11 | 8 | 18 | 12 | 8 | 22 | 12 | 10 | 25 | 19 | 12 | 31 | 24 | 20 | 21 | 18 | 6 | 21 | 18 | 9 |
| B16 | 16 | 9 | 7 | 15 | 10 | 7 | 27 | 16 | 12 | 26 | 18 | 16 | 32 | 26 | 17 | 20 | 15 | 7 | 19 | 15 | 7 |
| C1 | 6 | 5 | 4 | 5 | 3 | 3 | 16 | 11 | 6 | 23 | 12 | 8 | 20 | 17 | 12 | 8 | 5 | 3 | 12 | 7 | 4 |
| C2 | 5 | 5 | 3 | 5 | 3 | 2 | 14 | 9 | 6 | 22 | 14 | 11 | 19 | 11 | 9 | 8 | 5 | 3 | 11 | 8 | 4 |
| C3 | 7 | 4 | 3 | 4 | 3 | 2 | 12 | 8 | 5 | 26 | 13 | 11 | 17 | 11 | 8 | 6 | 5 | 2 | 10 | 5 | 4 |
| C4 | 10 | 6 | 4 | 8 | 4 | 2 | 15 | 11 | 7 | 21 | 13 | 10 | 21 | 10 | 8 | 10 | 5 | 4 | 11 | 7 | 4 |
| C5 | 11 | 5 | 4 | 9 | 5 | 4 | 12 | 10 | 6 | 19 | 10 | 8 | 22 | 10 | 8 | 9 | 5 | 3 | 14 | 7 | 4 |
| C6 | 8 | 5 | 3 | 6 | 4 | 2 | 13 | 9 | 6 | 21 | 8 | 9 | 18 | 8 | 6 | 6 | 3 | 3 | 9 | 5 | 3 |
| C7 | 13 | 6 | 4 | 7 | 2 | 3 | 12 | 9 | 7 | 24 | 17 | 14 | 21 | 8 | 9 | 8 | 4 | 3 | 9 | 4 | 3 |
| C8 | 15 | 10 | 4 | 13 | 9 | 4 | 17 | 9 | 8 | 23 | 14 | 10 | 22 | 15 | 9 | 15 | 10 | 5 | 19 | 12 | 5 |
| C9 | 16 | 8 | 4 | 12 | 8 | 5 | 14 | 7 | 6 | 23 | 15 | 14 | 25 | 12 | 10 | 13 | 7 | 3 | 12 | 9 | 4 |
| C10 | 13 | 9 | 4 | 11 | 8 | 3 | 15 | 7 | 7 | 25 | 9 | 8 | 22 | 15 | 8 | 12 | 8 | 4 | 11 | 7 | 3 |
| C11 | 10 | 7 | 3 | 10 | 7 | 2 | 14 | 7 | 7 | 23 | 19 | 11 | 21 | 11 | 7 | 14 | 7 | 3 | 10 | 6 | 3 |
| C12 | 8 | 6 | 3 | 9 | 5 | 2 | 10 | 6 | 6 | 21 | 8 | 6 | 19 | 9 | 7 | 10 | 6 | 3 | 10 | 6 | 4 |

* The unit of time for all the results presented in this table is ‘minutes’
